# Most functional outcomes are similar for men and women after hip fracture: a secondary analysis of the enhancing mobility after hip fracture trial

**DOI:** 10.1186/1471-2318-14-140

**Published:** 2014-12-18

**Authors:** Lynda M Woodward, Lindy Clemson, Anne M Moseley, Stephen R Lord, Ian D Cameron, Catherine Sherrington

**Affiliations:** Ageing, Work and Health Research Unit, Faculty of Health Sciences, The University of Sydney, Sydney, Australia; Musculoskeletal Division, The George Institute for Global Health, Sydney Medical School, The University of Sydney, Sydney, Australia; Australian Research Council Centre for Excellence in Population Ageing Research, Sydney, Australia; Neuroscience Research Australia, The University of New South Wales, Sydney, Australia; John Walsh Centre for Rehabilitation Research, Sydney Medical School, The University of Sydney, Sydney, Australia

**Keywords:** Gender, Hip fracture, Exercise, Rehabilitation

## Abstract

**Background:**

The impact of gender on functional outcomes after hip fracture is not known. We aimed to determine the extent to which gender influenced functional outcome and response to exercise in older people after hip fracture, and to determine if any differences persisted after adjusting for cognition, weight and age.

**Method:**

Secondary analysis of data from the Enhancing Mobility After Hip Fracture trial in which older people after hip fracture received either a lower or higher intensity exercise program. Functional outcomes included physical performance and self-reported measures. Regression models were used to compare genders at baseline, week 4 and week 16, with adjustment for baseline values, cognition, weight and age. Interaction terms were used to assess a differential impact of the intervention by gender.

**Results:**

Outcome data were available for 160 participants, 30 men (19%) and 130 women (81%) at baseline, with the withdrawal of 4 men (13%) and 6 women (5%) at week 16. There were no gender differences for any baseline measures or for most of the 19 functional outcome measures at weeks 4 and 16. At week 4 men performed better in knee extensor strength (2.1kg, 95% CI 0.6 to 3.7, p < 0.01). This difference did not persist after adjustment for body weight, however persisted after adjusting for baseline, cognition, and age (p = 0.038). At week 4, men performed better in coordinated stability (-10.0 error score, 95% CI -17.6 to -2.4, p=0.010) and this persisted after adjusting for baseline values only but not for cognition and age (p = 0.073). At week 16, men performed better in coordinated stability (-10.2 error score, 95% CI -18.4 to -1.9, p=0.016) and this persisted after adjusting only for cognitive impairment (p = 0.029) but not for age and baseline (p = 0.135). There was no indication of a differential impact of intervention type on the basis of gender.

**Conclusions:**

A few between gender differences were observed in strength and balance, however these appeared to be confounded by body weight, age and/or cognition.

**Electronic supplementary material:**

The online version of this article (doi:10.1186/1471-2318-14-140) contains supplementary material, which is available to authorized users.

## Background

Hip fracture is a major global health issue for older people, with the risk of hip fracture increasing with age [1]. The incidence of hip fracture continues to rise [2] due to population ageing [3], and in particular, to the increasing life expectancy of men [4]. Within the first six months of a hip fracture the mortality rate doubles [5]. Hip fractures are also associated with an increased risk of hospitalisation, institutionalisation and need for home assistance and decreased life expectancy [6]. Strength and balance exercises for older people after hip fracture can improve outcomes [7, 8], however the optimal nature of these programs is not well understood [9].

Men and women have been found to have different rehabilitation outcomes in other areas of health. For example, in older people undertaking exercise training for risk factors of cardiac disease, women had greater improvements with measures of body mass index and blood pressure compared with men [10]. Similarly, in older people with orthopaedic or neurological conditions undertaking exercise and balance training in aged care rehabilitation settings, older women improved more than older men on various measures including knee extensor strength [11] and reported pain levels [11, 12].

Several studies have shown outcome differences between men and women after hip fracture. Men have a tendency to have a higher mortality rate compared with women [13, 14], and men were more likely to require institutionalisation and an assistive device to mobilise [14]. The influence of gender on functional outcomes after participation in an exercise program in the older population after a hip fracture requires further investigation. There is limited research currently in this field, with one study finding no gender differences in Function Independence Measures (FIM) score after general rehabilitation participation following a hip fracture [15].

In order to investigate the impact of gender on functional outcome after hip fracture in the older population, we performed a secondary analysis on data from the Enhancing Mobility After Hip Fracture randomised controlled trial [16]. In the primary study, the authors investigated the effects of higher versus lower intensity balance, strength and walking exercises on rehabilitation inpatients after surgical fixation for a hip fracture [16]. The trial found no benefit or harm for the primary outcomes (knee extensor strength and gait speed) from undertaking a higher intensity exercise program in this population as a whole, however, those with a cognitive impairment recorded better outcomes after undertaking the higher intensity exercise program [16]. In this secondary analysis we aimed to determine the extent to which gender influenced functional outcome and the response to an exercise program after surgical fixation for a hip fracture, and to determine if any differences persisted after adjusting for cognitive deficits and age.

## Method

### Participants

A comprehensive description of the methods of the Enhancing Mobility After Hip Fracture trial have been reported elsewhere [16]. In brief, participants were recruited from three rehabilitation units in Sydney, Australia after surgical fixation for a fractured hip. Inclusion criteria included the ability to weight-bear (either fully or partially), participate in exercise, take four or more steps (either with or without assistance from a person and/or an assistive walking device), no medical contraindications to exercise, living at home or low-level residential care prior to having the hip fracture and planning to return to this living arrangement on discharge. People with more than four adjusted errors on the Short Portable Mental Status Questionnaire (SPMSQ) were included if they had a carer to supervisor the exercise program. Participants were classified as having cognitive impairment if they had three or more adjusted errors on the SPMSQ [17]. This work was approved by Hornsby, Ryde and Macquarie Hospitals’ Ethics Committee, the Royal Rehabilitation Centre Sydney Ethics Committee, South Western Sydney Area Health Service Research Ethics Committee, and The University of Sydney Human Research Ethics Committee.

After obtaining written informed consent, participants were randomly allocated into either the higher dose weight-bearing exercise group (HIGH) or the lower dose weight-bearing exercise group (LOW). Participants in the HIGH group performed exercises twice daily totalling 60 minutes of exercise for 16 weeks. Exercises included walking, stepping in different directions, repetitions of standing up and sitting down, tapping the foot onto a block, stepping onto and off a block. Participants in the LOW group received exercises represented that of usual care [18], and included exercises in supine and sitting plus some walking. Exercises were performed once daily totalling 30 minutes of exercise for 4 weeks.

### Functional outcome measures

Functional outcome measures included physical performance and self-reported measures. The physical performance measures included tests of strength (knee extensor strength of the fractured lower limb [19]), balance (maximum balance range, body sway, step test, lateral stability measurement, coordinated stability, and choice stepping reaction time [20–25]) and mobility (six metre walking speed [19], use of assistive walking device, sit to stand time [26], Physical Performance and Mobility Examination (PPME) [27] and Barthel Index [28]). Self-reported measures were fear of falling (Modified Falls Efficacy Scale (MFES) [29]), quality of life (EQ5D [30]), strength, balance, mobility, pain and overall health. Measures were undertaken at baseline, after 4 weeks and again after 16 weeks of the exercise program. Knee extension strength and walking speed were specified as the primary outcomes.

### Statistical analyses

We investigated between-gender differences with linear regression for continuous data, and logistic regression for dichotomised categorical data. HIGH and LOW groups were combined to determine between-gender differences at each time point. HIGH and LOW groups were separated and group x gender interaction terms were added to the models to assess whether there was a differential effect of group allocation on the basis of gender (i.e., an interaction between group and gender). Separate analyses were performed for baseline, week 4 and week 16. Analyses of week 4 and week 16 data were also adjusted for baseline values of the outcome considered. To account for expected gender differences in height and weight, the knee extension strength variable was also adjusted for body weight by multiplying the values obtained for each individual by the average weight of all participants divided by that participant’s weight [31]. Likewise, coordinated stability variable was adjusted for height by multiplying the values obtained by the participant’s height divided by the average height of all participants [23]. Cognition (dichotomised as 3 or more errors on the SPMSQ) and age (in years) were entered as covariants in the models to determine whether adjusting for cognition and/or age had an impact on the results. All analyses were completed using SPSS statistical software package and used the enter method. For the multivariable models, linear regression assumptions were checked by inspection of the distribution of the residuals.

## Results

### Participants

Baseline characteristics of participants are summarised in Table 1. One hundred and sixty participants were randomised, 30 men (19%) and 130 women (81%). There were 15 men and 65 women in each of the HIGH and LOW groups. The week 16 assessment was not completed by 4 men (13%; n = 3 HIGH; n = 1 LOW) and 6 women (5%; n = 4 HIGH; n = 2 LOW). Compared with the women, the men were significantly heavier (68.7 kg versus 58.5 kg, p < 0.01), significantly taller (168.3 cm versus 157.6 cm, p < 0.01), and significantly less likely to live in a hostel (3.3% versus 21.5%, p = 0.020). There were no other statistically significant or clinically important differences between genders for the baseline characteristics.

**Table 1:** **Participant characteristics at baseline by gender**

	Women (n = 130)	Men (n = 30)	Mean differences (95% CI) or chi squared, p
Group (HIGH:LOW)	65:65	15:15	-
Age at fracture (years), mean (SD)	84.4 (6.7)	81.7 (10.6)	2.7 (-0.4 to 5.7), 0.083
Height (cm), mean (SD)	157.6 (7.7)	168.3 (14.5)	-10.7 (-14.4 to -7.0), **p < 0.01**
Mass (kg), mean (SD)	58.5 (12.0)	67.8 (15.0)	-9.3 (-14.4 to -4.3), **p < 0.01**
Pre-fracture Barthel Index, mean (SD)	93.9 (9.0)	96.2 (9.3)	-2.3 (-5.9 to 1.4), 0.217
Pre-fracture falls, mean (SD)	1.7 (1.5)	2.3 (2.3)	-0.6 (-1.3 to 0.0), 0.066
Pre-fracture co-morbidities, mean (SD)	3.8 (1.8)	4.1 (2.1)	-0.3 (-1.1 to 0.4), 0.384
Pre-fracture services, mean (SD)	1.1 (1.4)	1.1 (1.3)	0.1 (-0.5 to 0.6), 0.859
Medications, mean (SD)	6.7 (2.7)	5.8 (2.5)	0.9 (-0.2 to 2.0), 0.094
Hostel dwelling, n (%)	28 (21.5)	1 (3.3)	5.4, **p = 0.020**
Type of pre-fracture walking aid,			
No aids or using walking stick or crutches, n (%)	104 (80)	26 (86.7)	0.7, p = 0.399
All other aids, n (%)	26 (20)	4 (13.3)	
Left hip fracture, n (%)	77 (59.2)	18 (60)	0.006, p = 0.938
Type of fracture,			
Intra-capsular, n (%)	63 (49.2)*	15 (50)	0.006, p = 0.939
Trochanteric, n (%)	65 (50.8)*	15 (50)	
Type of surgery,			
Bone screw, plate or other, n (%)	79 (60.8)	17 (56.7)	0.1, p = 0.738
Arthroplasty, n (%)	51 (39.2)	13 (43.3)	
Short Portable Mental Status Questionnaire ≥3 adjusted errors, n (%)	48 (36.9)	6 (20)	3.2, p = 0.077

Bold indicates p < 0.05; *n = 128.

### Outcomes

There were no statistically significant differences between men and women for any of the physical performance measures (Table 2) or self-reported measures (Table 3) at baseline.

**Table 2:** **Between-gender differences combining HIGH and LOW group participants for physical performance measures, including baseline adjustment**

	Baseline (n = 160)	Week 4 (n = 158)	Week 4-baseline (n = 158)	Week 16 (n = 150)	Week 16-baseline (n = 150)
**Strength measures**					
Knee extensor strength, kg	1.0 (-0.4 to 2.3), 0.151^†^	2.1 (0.6 to 3.7), **0.008** ^†^	1.8 (0.3 to 3.2), **0.020** ^†^	1.8 (-0.2 to 3.8), 0.070^†^	1.4 (-0.5 to 3.3), 0.149^†^
**Mobility measures**					
Walk speed, m/s	0.04 (-0.04 to 0.1), 0.332	0.04 (-0.05 to 0.1), 0.387	0.02 (-0.06 to 0.1), 0.634	0.06 (-0.7 to 0.2), 0.364	0.02 (-0.1 to 0.1), 0.726
PPME	-0.2 (-0.9 to 0.5), 0.555	0.5 (-0.3 to 1.2), 0.226^†^	0.6 (-0.1 to 1.3), 0.100^†^	0.4 (-0.6 to 1.4), 0.448^†^	0.4 (-0.5 to 1.4), 0.383^†^
Barthel index	3.4 (-2.1 to 8.9), 0.224^†^	1.5 (-3.6 to 6.7), 0.562^†^	0.4 (-4.4 to 5.2), 0.881^†^	2.4 (-3.9 to 8.6), 0.457^†^	1.0 (-4.7 to 6.8), 0.720^†^
Sit to stand, stand-ups/sec	0.01 (-0.02 to 0.04), 0.434^†^	-0.007 (-0.6 to 0.5), 0.797	-0.01 (-0.06 to 0.04), 0.591	-0.03 (-0.09 to 0.02), 0.259	-0.05 (-0.1 to 0.004), 0.071
No aids, using walking stick or crutches*	2.1 (0.6 to 7.2), 0.255^†^	2.0 (0.9 to 4.4), 0.109^†^	1.8 (0.7 to 4.5), 0.206^†^	2.0 (0.8 to 5.2), 0.140^†^	1.9 (0.7 to 4.8), 0.205^†^
**Balance measures**					
Maximum balance range, mm	4.2 (-10.5 to 18.9), 0.572^†^	4.6 (-11.9 to 21.1), 0.584^†^	2.7 (-12.9 to 18.2), 0.735^†^	12.2 (-7.2 to 31.6), 0.217^†^	8.9 (-9.7 to 27.5), 0.343^†^
Body sway total, mm	29.5 (-55.9 to 115), 0.496	21.5 (-61.1 to 104), 0.608	10.0 (-65.1 to 85.0), 0.793	-6.6 (-86.9 to 73.7), 0.872^†^	-8.1 (-78.8 to 62.7), 0.822^†^
Step test	-0.4 (-1.2 to 0.4), 0.337	0.3 (-1.6 to 2.2), 0.733^†^	0.5 (-1.4 to 2.4), 0.622^†^	-0.5 (-2.5 to 1.6), 0.661	-0.5 (-2.5 to 1.7), 0.675
Lateral stability, mm	4.9 (-3.5 to 13.3), 0.247	-0.5 (-9.8 to 8.8), 0.916^†^	-1.9 (-11.0 to 7.1), 0.671^†^	4.7 (-3.8 to 13.2), 0.278	3.0 (-5.1 to 11.0), 0.462
Coordinated stability	-4.8 (-12.0 to 2.5), 0.194^†^	-10.0 (-17.6 to -2.4), **0.010** ^†^	-6.7 (-12.4 to -1.0), **0.023** ^†^	-10.2 (-18.4 to -1.9), **0.016** ^†^	-6.3 (-13.1 to 0.6), 0.074^†^
Choice stepping reaction time, sec	N/A	-600 (-1484 to 285), 0.182^†§^	N/A	-400 (-1375 to 574), 0.418^†‡^	N/A

Results as mean (95% CI), p unless *indicating results as odds ratio (CI 95%), p; bold indicates p < 0.05; PPME = Physical performance and mobility examination; ^†^indicates men performed better than women; ^‡^n = 148; ^§^n = 146; N/A = not assessed.

**Table 3:** **Between-gender differences combining HIGH and LOW group participants for self-reported measures, including baseline adjustment**

	Baseline (n = 160)	Week 4 (n = 158)	Week 4-baseline (n = 158)	Week 16 (n = 150)	Week 16-baseline (n = 150)
Self-rated mobility as good*	1.2 (0.5 to 3.4), 0.690^†^	1.0 (0.5 to 2.4), 0.940^†^	1.0 (0.4 to 2.3), 0.999^†^	0.5 (0.2 to 1.1), 0.082^†∫^	0.5 (0.2 to 1.1), 0.078^†∫^
Self-rated strength as good*	0.8 (0.2 to 2.4), 0.632^†^	0.4 (0.1 to 1.3), 0.136^†^	0.4 (0.1 to 1.4), 0.155^†^	0.6 (0.3 to 1.5), 0.310^†∫^	0.7 (0.3 to 1.7), 0.387^†∫^
Self-rated balance as good*	0.9 (0.4 to 2.0), 0.718^†^	1.0 (0.4 to 2.4), 0.940^†^	1.0 (0.4 to 2.6), 0.953^†^	0.8 (0.3 to 1.9), 0.611^†∫^	0.9 (0.4 to 2.0), 0.711^†∫^
Self-rated pain as none or slight*	0.8 (0.3 to 1.9), 0.602^†^	0.7 (0.3 to 1.6), 0.450^†^	0.8 (0.3 to 1.7), 0.505^†^	1.0 (0.4 to 2.5), 0.920^†^	1.1 (0.5 to 2.6), 0.887^†^
Self-rated health as worse*	1.0 (0.4 to 2.1), 0.899	0.9 (0.4 to 2.0), 0.772^§^	0.9 (0.4 to 2.0), 0.785^§^	0.7 (0.3 to 1.9), 0.476^∫^	0.8 (0.3 to 2.1), 0.605^∫^
Modified falls efficacy scale	10.3 (-2.3 to 22.8), 0.109^†‡^	8.4 (-3.8 to 20.7), 0.174^†§^	3.7 (-7.3 to 14.6), 0.507^†ǁ^	4.7 (-9.8 to 19.3), 0.520^†∫^	-0.7 (-13.8 to 12.4), 0.916^†^**
EQ5D	0.02 (-0.09 to 0.1), 0.765	0.04 (-0.07 to 0.2), 0.510	0.03 (-0.07 to 0.1), 0.535	-0.03 (-0.2 to 0.09), 0.610^†∫^	-0.04 (-0.2 to 0.08), 0.529^†∫^

Results as mean (95% CI), p unless *indicating results as odds ratio (CI 95%), p; ^†^indicates men performed better than women; ^‡^n = 158; ^§^n = 157; ^ǁ^n = 155; ^∫^n = 149; **n = 146.

Men were significantly stronger than women in the raw measure of knee extensor strength at week 4 (2.1 kg, 95% CI 0.6 to 3.7, p = 0.008) and week 4 adjusted for baseline (1.8 kg, 95% CI 0.3 to 3.2, p = 0.020) (Table 2). Men were also stronger than women at week 16 but this difference was not statistically significant (1.8 kg, 95% CI -0.2 to 3.8, p = 0.070; adjusted for baseline, 1.4 kg, 95% CI -0.5 to 3.3, p = 0.149). However using the body weight adjusted variable, the impact of gender on knee extension strength was no longer statistically significant at these time points (week 4, 1.0 kg, 95% CI -0.5 to 2.5, p = 0.0187; week 4 adjusted for baseline, 1.1 kg, 95% CI -0.4 to 2.5, p = 0.144) (see Additional file 1).

Men performed significantly better than women on tests of coordinated stability at week 4 (-10.0, 95% CI -17.6 to -2.4, p = 0.010), week 4 adjusted for baseline (-6.7, 95% CI -12.4 to -1.0, p = 0.023), and week 16 (-10.2, 95% CI -18.4 to -1.9, p = 0.016) (Table 2). Using the height adjusted variable, the impact of gender on coordinated stability remained significant at week 4 (-8.1, 95% CI -15.8 to -0.3, p = 0.041), week 4 adjusted for baseline (-6.1, 95% CI -11.9 to -0.3, p = 0.040), and at week 16 (-8.6, 95% CI -16.9 to -0.4, p = 0.040) (see Additional file 1).

There were no statistically significant differences between men and women for any of the other physical performance measures at week 4 or week 16 (Table 2). Likewise, there were no statistically significant differences between men and women for any of the self-reported measures at week 4 or week 16 (Table 3).

The between-gender differences in knee extensor strength in unadjusted analyses all remained statistically significant after adjusting for cognition and age, however, the between-gender differences in coordinated stability in unadjusted analyses only remained significant after adjusting for cognition and age in separate models. Once age and cognition were combined within the same model, the between-gender differences in coordinated stability in unadjusted analyses were no longer statistically significant at any time point (Table 4). After adjusting for baseline values, these multivariable model explained 14% and 14% of variability in knee extension strength, and 50% and 36% of variability in coordinated stability, at week 4 and week 16 respectively.

**Table 4:** **Knee extension strength and coordinated stability between-gender differences adjusting for cognition and age**

	Baseline (n = 160)	Week 4 (n = 158)	Week 4-baseline (n = 158)	Week 16 (n = 150)	Week 16-baseline (n = 150)
**Knee extensor strength, kg**					
Cognition	-1.0 (-2.1 to 0.1), 0.073	-1.0 (-2.3 to 0.3), 0.137	-0.6 (-1.9 to 0.6), 0.322	-1.1 (-2.7 to 0.5), 0.180	-0.6 (-2.2 to 0.9), 0.417
Cognition controlling for gender	-0.9 (-2.0 to 0.2), 0.108	-0.8 (-2.1 to 0.5), 0.244	-0.5 (-1.7 to 0.8), 0.468	-0.9 (-2.5 to 0.7), 0.245	-0.5 (-2.1 to 1.0), 0.493
Gender controlling for cognition and age	0.9 (-0.5 to 2.2), 0.216	1.9 (0.3 to 3.5), **0.018**	1.6 (0.09 to 3.1), **0.038**	1.4 (-0.5 to 3.4), 0.154	1.0 (-0.9 to 2.9), 0.287
**Coordinated stability**					
Cognition	6.4 (0.4 to 12.3), **0.036**	11.7 (5.5 to 17.9), **0.000**	7.4 (2.6 to 12.1), **0.003**	9.0 (2.4 to 15.5), **0.008**	4.9 (-0.6 to 10.4), 0.080
Age	0.6 (0.3 to 1.0), **0.001**	0.9 (0.5 to 1.2), **0.000**	0.4 (0.1 to 0.7), **0.006**	0.7 (0.4 to 1.1), **0.000**	0.4 (0.03 to 0.7), **0.035**
Cognition controlling for gender	5.9 (-0.07 to 11.9), 0.053	10.8 (4.7 to 17.0), **0.001**	6.8 (2.1 to 11.6), **0.005**	8.2 (1.7 to 14.7), **0.014**	4.5 (-1.0 to 10.0), 0.105
Age controlling for gender	0.6 (0.3 to 1.0), **0.001**	0.8 (0.4 to 1.2), **0.000**	0.4 (0.09 to 0.7), **0.011**	0.7 (0.3 to 1.1), **0.001**	0.3 (-0.003 to 0.7), 0.052
Gender controlling for cognition	-3.8 (-11.1 to 3.5), 0.304	-8.3 (-15.7 to -0.8), **0.029**	-5.7 (-11.3 to -0.08), **0.047**	-9.1 (-17.2 to -1.0), **0.029**	-5.8 (-12.6 to 1.1), 0.097
Gender controlling for age	-3.1 (-10.2 to 4.0), 0.387	-7.9 (-15.2 to -0.6), **0.034**	-5.8 (-11.5 to -0.2), **0.043**	-8.2 (-16.2 to -0.2), **0.045**	-5.5 (-12.4 to 1.3), 0.112
Gender controlling for cognition and age	-2.5 (-9.6 to 4.6), 0.488	-6.7 (-13.8 to 0.5), 0.066	-5.1 (-10.7 to 0.5), 0.073	-7.5 (-15.5 to 0.4), 0.063	-5.2 (-12.0 to 1.6), 0.135

Bold indicates statistical significance p < 0.05; cognition output from univariate model with cognition as the predictor; age output from univariate model with age as the predictor; cognition controlling for gender, age controlling for gender, gender controlling for cognition, gender controlling for age, and gender controlling for cognition and age outputs from multivariate model with cognition, gender and age as predictors.

There was no evidence of a differential effect for any of the physical performance measures and self-reported measures in relation to between-group differences based on gender (see Additional file 2).

## Discussion

Our study showed that, among older people participating in an exercise program after surgical fixation for a hip fracture, gender impacted on only one of six measures of balance, namely coordinated stability. Cognition did not affect these between-gender differences. There were no between-gender differences for any of the other physical performance measures or self-reported measures tested and no evidence of a differential effect of the higher intensity or lower intensity interventions based on gender.

Men performed better in tests of knee extensor strength compared with women after 4 weeks of the exercise program, however, these between-gender differences in knee extension strength were no longer evident once adjusted for body weight. Hence, we suggest that future studies should adjust for body weight when determining between-gender differences for this measure. It is unclear whether it was body weight *per se* that differentiated between men and women or whether body weight was a marker of muscle mass and strength. Further studies could investigate this issue.

Men also performed better in tests of coordinated stability after 4 and 16 weeks of the exercise program. Coordinated stability tests participants’ balance control at the limits of their base of support in a way that is both controlled and coordinated [20, 25] so it is likely to be a marker of ease of performance of daily tasks that require control of the body’s movements in space. The gender differences in coordinated stability remained significant after adjusting for height. As there were no between-gender differences in coordinated stability at baseline and as the 4-week between-gender differences were evident after adjustment for baseline performance these results suggest that men may recover more quickly in the early stages of an exercise program. This difference between men and women regarding coordinated stability outcome was interesting as none of the other balance outcomes showed any differences between men and women. The clinical relevance of this gender difference is unknown and may be a chance finding warranting the need for further research. It is interesting to note that the difference in coordinated stability measures between fallers and non-fallers has been reported to be only three points [32].

To our knowledge no previous studies have investigated gender differences in physical function with strength and balance exercise programs for hip fracture. A previous study found men and women to have similar rehabilitation outcomes measured with FIM scores [15] but the use of physical performance measures in our study may have enabled the detection of subtler differences. Yet since our study found no between-gender differences for the majority of measures tested, it is possible that the co-ordinated stability finding is due to chance.

The major weakness of this analysis is the unequal number of men and women with a relatively small number of men. As this analysis showed gender differences for one of the functional outcomes we suggest that further research with larger samples is warranted. It is also possible that the gender difference for the single balance measure is a chance finding due to multiple testing. This also suggests the need for further investigation of this issue.

There was no indication of a differential effect of the intervention. However men and women might prefer different types of interventions. Further research with larger samples could also investigate whether different approaches to intervention have differential impacts in men and women and whether impressions of interventions are different between the genders.

## Conclusion

There were no gender differences in physical performance or self-rated measures among a sample of older people prior to commencing the strength and balance exercise programs. No differences were observed for the majority of the functional outcomes tested and no differential effect of intervention was detected. This study found gender differences in recovery after hip fracture for one measure of balance performance, namely coordinated stability. Some observed between gender differences appeared to be confounded by body weight, age and/or cognition, highlighting the need for such adjustment in future studies. The impact of gender on functional outcome for older people affected by hip fracture deserves further attention as hip fracture is a common consequence of falls.

## Electronic supplementary material

Additional file 1: **Knee extensor strength and coordinated stability measures adjusted for weight or height.** This table shows the results of knee extensor strength analysis controlling for weight and coordinated stability analysis controlling for height for all time points and adjusting for baseline. (XLSX 10 KB)

Additional file 2: **Between-group differences separated for gender and interactions between gender and group.** This table shows the analysis for all physical performance measures and self-rated measures for between-group differences for men and women at week 16 and also group x gender interaction at week 16. (XLSX 16 KB)

## Abbreviations

FIM: Function independence measures
SPMSQ: Short portable mental status questionnaire
HIGH: High dose weight-bearing exercise group
LOW: The low dose weight-bearing exercise group
PPME: Performance and mobility examination
MFES: Modified falls efficacy scale.
